# Relationship of insight with medication adherence and the impact on outcomes in patients with schizophrenia and bipolar disorder: results from a 1-year European outpatient observational study

**DOI:** 10.1186/s12888-015-0560-4

**Published:** 2015-08-05

**Authors:** Diego Novick, William Montgomery, Tamas Treuer, Jaume Aguado, Susanne Kraemer, Josep Maria Haro

**Affiliations:** Global Health Outcomes Research, Lilly Research Centre, Eli Lilly and Company, Erl Wood Manor, Windlesham, Surrey GU20 6PH UK; Eli Lilly Australia Pty Ltd, West Ryde, Australia; Eli Lilly and Company, Budapest, Hungary; Parc Sanitari Sant Joan de Déu, CIBERSAM, Universitat de Barcelona, Barcelona, Spain; Lilly Deutschland GmbH, Bad Homburg, Germany

**Keywords:** Insight, Medication adherence, Olanzapine, Schizophrenia, Bipolar disorder

## Abstract

**Background:**

Many patients with schizophrenia and bipolar disorder have impaired insight and low medication adherence. The aim of this post hoc analysis was to explore the relationship between insight and medication adherence.

**Methods:**

We included 903 patients with schizophrenia or bipolar disorder who participated in an observational study conducted in Europe on the outcomes of patients treated with two oral formulations of olanzapine over a 1-year period. Evaluations included Clinical Global Impression (CGI), Global Assessment of Functioning (GAF), insight (Scale to Assess Unawareness of Mental Disorder, SUMD) medication adherence (Medication Adherence Rating Scale, MARS), and therapeutic alliance (Working Alliance Inventory, WAI).

**Results:**

Medication adherence was higher in bipolar patients (mean MARS score (SD) 6.5 (2.8) versus 5.8 (2.7) in schizophrenia; *p* < 0.001). Patients with schizophrenia had lower insight (i.e., SUMD item 1, unawareness of mental disorder, mean (SD) of 2.5 (1.3) in schizophrenia versus 1.9 (1.2) in bipolar, *p* < 0.001). Better insight was associated with higher adherence (Spearman Correlation Coefficient, SCC, ranging from 0.39 to 0.49 for the three SUMD general items, *p* < 0.0001 in all cases). Higher insight was related to a stronger therapeutic alliance (SCC ranging from 0.38 to 0.48, *p* < 0.0001). A path analysis revealed a positive impact of insight on adherence and alliance and that stronger alliance was related to lower clinical severity (lower CGI score).

**Conclusion:**

Insight and adherence were found to be closely related. Insight impacts on the therapeutic alliance with mental health professionals. These factors are associated to treatment outcomes.

## Background

Although a number of psychotropic medications are available and effective to treat the manifestations of schizophrenia and bipolar disorder, many patients do not actually benefit from them due to low adherence with medication regimens. Rates of low adherence have been reported to be as high as two-thirds in patients with schizophrenia [1]. Young et al. also concluded that 40 % of patients treated with conventional antipsychotics stop taking their medication within one year [2]. Non-adherence is a particular challenge in schizophrenia due to the illness association with social isolation, stigma, and co-morbid substance misuse [3]. Rates of low adherence are also high in patients with bipolar disorder: in a recent study of 303 patients, 69 % of patients showed suboptimal adherence [4, 5]. Low adherence rates have been shown to be one of the main causes of relapse and hospitalization [6, 7].

In a recent review in patients with schizophrenia, illness insight and positive attitude to medication were the only factors consistently associated with better adherence. Contradictory results were found for other characteristics such as socio-demographics, symptom severity and side effects [8]. Poor insight is a core attribute of schizophrenia, occurring in 57–98 % of patients [9, 10] and it is modulated by positive symptom severity [11]. Lack of insight has also been reported as one of the most relevant factors associated with medication adherence in patients with bipolar disorder [12].

Insight also impacts on the therapeutic relationship [7]. In a small sample of inpatients with schizophrenia or schizoaffective disorder, Misdhahi et al. found that a weak therapeutic alliance and low insight were associated with poor adherence in patients with schizophrenia or schizoaffective disorder [13]. Some studies have actually studied the relationship between insight, therapeutic relationship and adherence [14]. However, they were limited due to small samples of patients or did not include both in- and outpatients.

Up to now little emphasis has been given to strategies to increase insight in patients with severe mental disorders. Pijnenbourg et al., in a literature review on treatment for impaired insight in psychosis, confirmed that insight is a potential therapeutic target and that it is amenable to improvement. Comprehensive intervention programmes consisting of multiple components may be particularly promising [15].

In spite of the increasing research into insight, its causes and its impact on the course of schizophrenia, many issues remain unanswered, partly due to the inconsistent definitions of insight but also due to contradictory results about the impact of insight on patient outcomes [16]. The aim of this post-hoc analysis was to explore the relationship between insight, therapeutic relationship and medication adherence, and their impact on the outcomes of patients with schizophrenia or bipolar disorder. We hypothesize that lower insight will result in both a lower medication adherence and worse therapeutic relationship. Patients with lower insight will also have worse outcomes, mostly mediated by the effect of low adherence.

## Methods

### Study design

Data for this post hoc analysis were obtained from a prospective, observational (non-interventional), naturalistic, multicentre, multi-country study designed to compare medication adherence between two oral forms of olanzapine (either oro-dispersible or standard coated tablets) in patients with schizophrenia or bipolar disorder. Patients were followed for 1 year, with up to five study visits at approximately 3-month (±1 month) intervals. Data collection occurred when patients attended for their regular clinic visits. To reduce selection bias, each participating psychiatrist was asked to enroll consecutively, up to eight eligible patients.

### Patients

Patients were eligible for enrolment if they met the following entry criteria: adult patients diagnosed with schizophrenia or any type of bipolar disorder and for whom their physician decided to begin antipsychotic treatment with olanzapine (either oro-dispersible or standard coated tablet), according to the approved marketing authorization, within the last 45 days (either as treatment initiation or as switch from another antipsychotic). A total of 903 patients with schizophrenia or bipolar disorder from outpatient or hospital settings were enrolled in this study between April 2007 to April 2008, and the last study visit occurred in May 2009. The protocol did not restrict use to antipsychotic monotherapy or combination therapy. All treatment decisions were made at the discretion of the treating physician and patient, including the choice of olanzapine formulation.

All patients provided written, informed consent. The study was approved by ethical review boards as required by local law and was conducted in accordance with the ethical principles that have their origin in the Declaration of Helsinki. The specific ethics committees that approved the study are: (1) Comité consultatif sur le traitement de l'information en matière de recherche dans le domaine de la santé (France); (2) Landesärztekammer Hessen Ethik-Kommission (Germany); (3) Papageorgiou Regional General Hospital of Thessaloniki (Greece). Further details about the study have been published previously [17].

### Clinical assessments

Assessments were conducted by participating psychiatrists or their designees. All investigators participated in a start-up meeting in which training in the study procedures and questionnaire administration were provided. No formal assessment of inter-rater agreement was performed, however the included questionnaires and scales are commonly used in clinical practice.

Assessments included clinical severity, global functioning, adherence, insight and the therapeutic alliance. Clinical severity was measured with the Clinical Global Impression (CGI) scale for Bipolar Disorder (CGI-BP) [18] or Schizophrenia (CGI-SCH) [19]. The Global Assessment of Functioning Scale (GAF) was employed to measure functioning [20]. Medication adherence was estimated using the Medication Adherence Rating Scale (MARS), a 10-item self-reported measure ranging from 0 to 10 with a high score being associated with better adherence [21]. Insight was measured with the Scale to Assess Unawareness of Mental Disorder (SUMD) [22]. The analyses included here report on the first three items of the SUMD scale, which rate the unawareness of illness regarding having a mental disorder (SUM1), response to medication (SUM2) and the patient’s perception of the need for medication (SUM3). The scale is rated from 1 to 5 with higher ratings indicating higher unawareness/lower insight. Therapeutic alliance was assessed with the Working Alliance Inventory (WAI) [23]. This is a physician-reported scale that describes the relationship of the physician with the patient. The scale results range from 36 (minimum) to 252 (maximum), and higher ratings indicate a better relationship.

### Statistical analysis

Overall baseline patient characteristics by diagnosis were described and compared using the Chi-square test (for categorical variables) or Kruskal-Wallis test (for continuous variables). Spearman Correlation Coefficients (SCC) were employed to assess the associations between variables at baseline and between the changes from baseline to endpoint. The variables of the change scores were computed as the difference between baseline and endpoint in a way that greater difference means more improvement. Linear regression models were used to assess the effect of baseline insight components (separately) on outcomes at endpoint, adjusting for significant covariates. Covariates for the models were selected based on clinical criteria and descriptive analyses. Models including and not including the baseline rating of the outcome measure were fitted. A path analysis was used to understand the relationship between insight, adherence, therapeutic alliance and outcomes. The fit indices that were calculated included the root mean squared error (RMSEA) (good fit if <0.08), comparative fit index (CFI) (good fit if >0.95) and standardized root mean square residual (SRMR) (good fit if <0.05). The statistical analysis was carried out using SAS software (SAS Institute, Cary, NC, USA), version 9.3 and M-Plus 7.2.

## Results

### Patient demographics and clinical characteristics at baseline

Out of the 903 enrolled outpatients, 612 (67.8 %) had been diagnosed with schizophrenia and 291 (32.2 %) with bipolar disorder. A total of 493 patients (54.6 %) received orodispersible olanzapine tablets, whereas 410 (45.4 %) received standard coated olanzapine tablets, with similar proportions of the different formulations used in both diagnostic groups. Most patients (*n* = 735, 81.4 %) were treated in private ambulatory settings, while the remainder (*n* = 168, 18.6 %) came from hospital ambulatory settings. Table 1 presents further patient characteristics. The bipolar patient group were older and had a higher proportion of women. There were no differences in the psychiatric care setting between the two diagnostic groups. Compared with the schizophrenia patients, bipolar patients appeared to have significantly better general functioning, as shown by higher GAF scores. Medication adherence and insight were also higher in the bipolar patients.

**Table 1:** Baseline demographics and clinical characteristics of patients

Characteristics	Overall	Schizophrenia	Bipolar	*p*
	(*n* = 903)	(*n* = 612)	(*n* = 291)	
Gender, *n* (%) females	405 (44.9)	231 (37.7)	174 (59.8)	<0.001
Age (years); mean (SD)	40.9 (13.1)	39.2 (12.7)	44.6 (13.1)	<0.001
Insight				
SUM1 total score, mean (SD)	2.3 (1.3)	2.5 (1.3)	1.9 (1.2)	<0.001
SUM2 total score, mean (SD)	2.1 (1.2)	2.3 (1.3)	1.9 (1.1)	<0.001
SUM3 total score, mean (SD)	2.0 (1.0)	2.1 (1.0)	1.8 (1.0)	<0.001
Medication adherence				
MARS total score, mean (SD)	6.0 (2.8)	5.8 (2.7)	6.5 (2.8)	<0.001
Therapeutic alliance				
WAI total score, mean (SD)	188.5 (27.0)	185.0 (27.4)	195.9 (24.5)	<0.001
Clinical severity				
CGI total score, mean (SD)	4.0 (1.2)	4.0 (1.1)	3.9 (1.4)	0.305
Global functioning				
GAF total score, mean (SD)	54.0 (16.0)	51.9 (15.7)	58.4 (15.6)	<0.001

*CGI* clinical global impression, *GAF* global assessment of functioning, *MARS* medication adherence rating scale, *SUM1* unawareness of having a mental disorder, *SUM2* unawareness of response to medication, *SUM3* unawareness of the need of medication, *WAI* working alliance inventory

At baseline, patients with higher insight into their mental disorder were associated with higher medication adherence scores (SCC between each item of the SUMD and the MARSS ranged from −0.39 to −0.49, *p* < 0.0001 in all cases; Table 2) and a stronger therapeutic alliance (SCC between each item of the SUMD and the WAI score ranged from −0.38 to −0.48, *p* < 0.0001; Table 2). Higher insight at baseline was also associated with milder clinical severity (SCC between the SUMD items and the CGI score ranged from 0.29 to 0.39, *p* < 0.0001; Table 2) and better global functioning (SCC between the items of the SUMD and the GAF score ranged from −0.31 to −0.34, *p* < 0.0001; Table 2). A better patient–physician relationship was also associated with stronger medication adherence, superior global functioning, and a milder clinical severity of the mental illness.

**Table 2:** Correlation between insight, medication adherence, therapeutic working alliance, CGI and GAF at baseline (all patients)

	SUM1	SUM2	SUM3	MARS	WAI	CGI
SUM1						
SUM2	0.72^#^					
SUM3	0.71^#^	0.78^#^				
MARS	−0.39^#^	−0.46^#^	−0.49^#^			
WAI	−0.38^#^	−0.42^#^	−0.48^#^	0.49^#^		
CGI	0.32^#^	0.39^#^	0.29^#^	−0.28^#^	−0.16^#^	
GAF	−0.31^#^	−0.36^#^	−0.34^#^	0.26^#^	0.23^#^	−0.64^#^

*CGI* clinical global impression, *GAF* global assessment of functioning, *MARS* medication adherence rating scale, *SUM1* unawareness of having a mental disorder, *SUM2* unawareness of response to medication, *SUM3* unawareness of the need of medication, *WAI* working alliance inventory

### Change of patient insight, medication adherence, therapeutic alliance and clinical outcomes from baseline to endpoint

After 1 year of follow up, significant direct associations were found between an improvement in the patient’s awareness of their mental disorder (i.e., gain of insight) or an improvement in the patient–physician relationship and an increase in medication adherence, amelioration of global functioning and an improvement in the clinical outcome. Evidence of this was the positive correlation between the changes of the scores of all SUMD items or of the WAI scale with those of MARS, GAF, and CGI scores from baseline to endpoint (Table 3, all *p* < 0.001). At the same time, the improvement in patient insight was accompanied by a strengthening of the patient–physician relationship.

**Table 3:** Correlation amongst change in variables from baseline to endpoint (all patients)

	CGI	WAI	GAF	SUM1	SUM2	SUM3
MARS	0.33^#^	0.44^#^	0.41^#^	0.34^#^	0.40^#^	0.44^#^
CGI		0.29^#^	0.62^#^	0.33^#^	0.35^#^	0.32^#^
WAI			0.40^#^	0.31^#^	0.35^#^	0.37^#^
GAF				0.37^#^	0.40^#^	0.40^#^
SUM1					0.54^#^	0.53^#^
SUM2						0.63^#^

As higher ratings in some scales mean better outcomes and lower ratings in other scales mean better outcomes, the signs of Pearson correlation coefficients have been modified so that positive coefficients mean improvement in one variable is positively correlated with improvement in the other

*CGI* clinical global impression, *GAF* global assessment of functioning, *MARS* medication adherence rating scale, *SUM1* unawareness of having a mental disorder, *SUM2* unawareness of response to medication, *SUM3* unawareness of the need of medication, *WAI* working alliance inventory

Linear regression models were used to analyse the contribution of the different factors on clinical outcomes at the end of the study, as measured using the CGI and GAF, adjusting for covariates.

As shown in Table 4, the baseline ratings of the SUMD items were significantly correlated to the 1-year ratings of the CGI and GAF scales. MARS and WAI baseline ratings were also correlated to 1-year GAF ratings. The linear model which adjusted by baseline covariates and took into account the correlation among insight, therapeutic alliance and adherence (Table 3) showed that the baseline ratings of the first and second items of the SUMD scale (SUM1 and SUM2) were associated to the CGI and GAF at endpoint: worse insight (higher SUM1 and SUM2 scores) was associated to greater overall clinical severity (CGI score) and lower functioning (lower GAF score). Baseline therapeutic alliance was also associated to one year CGI and GAF scores, with better therapeutic alliance being associated to lower clinical severity and better functioning.

**Table 4:** Relationship between baseline insight, adherence and therapeutic relationship and CGI and GAF at endpoint (all patients)

	Univariate relationship (correlation coefficient)		Regression analysis coefficient (95 % CI)	
Baseline value	CGI	GAF	CGI	GAF
SUM1	0.12^#^	−0.18^#^	0.13 (0.06; 0.20)^#a^	−1.76 (−2.69; −0.84)^#a^
SUM2	0.14^#^	−0.16^#^	0.14 (0.07; 0.22)^#a^	−1.31 (−2.37; −0.25)*^a^
SUM3	0.09*	−0.13^#^	0.08 (−0.02; 0.17)^a^	−0.48 (−1.81; 0.84)^a^
MARS	−0.07	0.14^#^	0.02 (−0.01; 0.05)^b^	−0.02 (−0.47;0.44)^b^
WAI	−0.15^+^	0.23^#^	−0.01 (−0.01; −0.004)^#b^	0.13 (0.08; 0.17)^#b^

As higher ratings in some scales mean better outcomes and lower ratings in other scales mean better outcomes, the signs of Pearson correlation coefficients have been modified so that positive coefficients mean improvement in one variable is positively correlated with improvement in the other

*CGI* clinical global impression, *GAF* global assessment of functioning, *SUM1* unawareness of having a mental disorder, *SUM2* unawareness of response to medication, *SUM3* unawareness of the need of medication, *MARS* medication adherence rating scale, *WAI* working alliance inventory

^#^ *p* < 0.001

*p < 0 .05

^+^ *p* < 0.01

^a^Adjusted for country, age, sex, MARS and WAI

^b^Adjusted for country, age, sex, MARS, WAI and SUM1

A linear regression model including the baseline score of the outcome scale was also fitted and provided different results. In this model the relationship between the baseline rating of the SUMD items and the CGI and GAF at one year changed. Thus, SUM1 at baseline was not associated to CGI or GAF score at one year (regression coefficients 0.01 (95 % CI −0.06; 0.07) and 0.14 (−0.68, 0.97) respectively). SUM2 an SUM3 ratings were associated to endpoint GAF score (regression coefficients 1.05 (0.11; 1.99) and 2.49 (1.34; 3.64), respectively) but not to endpoint CGI score (regression coefficients −0.02 (−0.09; 0.06) and −0.06 (−0.15; 0.03), respectively). Finally, MARS and WAI score were both associated to endpoint CGI (regression coefficients 0.05 (0.02; 0.08) and −0.01 (−0.01; −0.004), respectively) and endpoint GAF scores (regression coefficients-0.41 (−0.80;-0.01) and (0.10 (0.06; 0.14 respectively) . R^2^ in all models values ranged from 0.09 to 0.10, which is low given the large number of factors impacting on the course of schizophrenia. However, it can be observed that the direction of the relationship changes when including the baseline rating of the covariate, which shows the high relationship among the variables.

### Model of interferences between different parameters

**Fig. 1:**
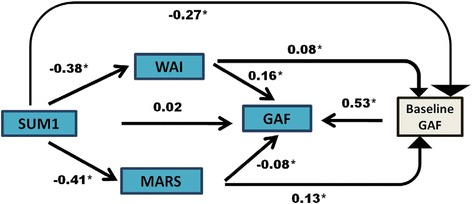
Path analyses relating insight, therapeutic working alliance, adherence and global assessment of functioning at endpoint (all patients)

**Fig. 2:**
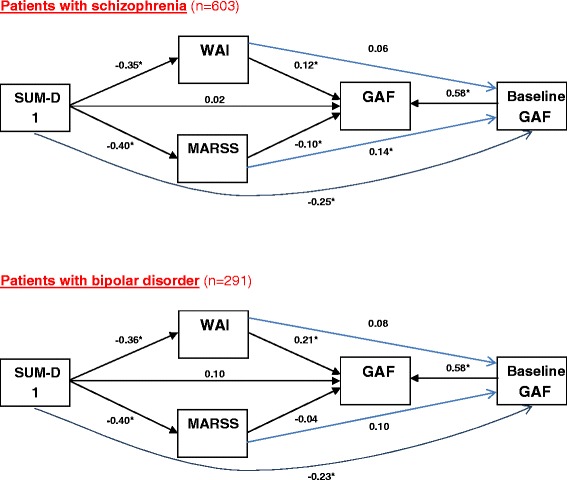
Path analyses relating insight, therapeutic working alliance, adherence and global assessment of functioning at endpoint, stratified by diagnostic group

Given the intense relationship between insight, therapeutic relationship and adherence, a path analysis was fitted to better understand these associations and how they influenced outcomes. Figure 1 presents the association of baseline ratings of the SUM1 (insight), WAI (therapeutic relationship) and MARS (adherence with medication) with GAF (functioning) at 1 year. As GAF at baseline was also highly correlated with GAF at endpoint, GAF at baseline was also included in the model. The model showed that, at baseline, lower insight was associated with lower adherence and a worse therapeutic relationship. The three variables were also associated with baseline GAF rating: better insight, better therapeutic relationship, and better adherence were all of them associated with better functioning. Therapeutic relationship, adherence and baseline GAF were also associated with 1-year GAF. The stronger relationship was between baseline and endpoint GAF score. A better therapeutic relationship was associated with better functioning, but greater adherence appeared to be associated with slightly poorer functioning. The model was saturated, and thus no fit indices could be calculated. We calculated the fit indices for the same model but not including the relationship between SUM1 and baseline GAF. In this case the parameters that evaluated model fit were good for CFI (0.97 which is >0.95) and SRMR (0.044, which is <0.05) and moderate for RMSEA (0.115, which is > 0.08). Additional analyses were conducted stratifying diagnostic group. Very similar results were found (Fig. 2).

## Discussion

Insight, therapeutic alliance with the treating psychiatrist and medication adherence are highly correlated in patients with schizophrenia and bipolar disorder. The three factors co-vary during the course of the disorder and an improvement in one is accompanied by improvements in the others. It is worth mentioning that these associations may be bi-directional: while insight may influence therapeutic relationship, therapeutic relationship may also influence insight. These factors are also related to patient outcomes. However, their independent relationship to patient outcomes is complex as it is difficult to disentangle the interrelationship between these variables in a prospective analysis of the course of the disorder.

Our results are consistent with previous studies that have analysed the relationship between these variables. For example, a cross-sectional study of patients with schizophrenia or bipolar disorder admitted due to acute exacerbation of their condition found that insight was related to patient attitude towards antipsychotic treatment at hospital discharge [24]. Another cross-sectional study with 38 inpatients who met ICD-10 criteria for schizophrenia or schizoaffective disorder showed that therapeutic alliance was significantly correlated with adherence and that a weak therapeutic alliance and low insight were associated with poor adherence [13]. Similar results have been reported for outpatients. A cross-sectional sample of 150 outpatients with schizophrenia analysed using a structural equation model, for example, found evidence for a mediational model in which insight contributed to medication adherence via patients’ perceived necessity of antipsychotics [14].

Few studies have included follow-up assessment. In a small study of 112 participants with schizophrenia or schizoaffective disorder followed for 6 months, Baloush-Kleinman et al. reported that adherent participants showed more insight into their illness, awareness of the need for medication and positive perceptions of trust in the patient–physician therapeutic alliance. In this study, structural equation modelling also showed that over 6 months, symptom severity, awareness for the need for medication and attitudes to medication predicted adherence [25].

Our study expands on previous findings by including a much larger sample of patients, a longer follow-up, and analysing the concurrent relationship between these factors. We have also found consistent results for the overall sample, and for patients with schizophrenia and bipolar disorder, respectively.

This study also raises some questions that deserve further exploration to fully understand the complex relationship between insight, therapeutic relationship, adherence and outcomes. For example, we found that baseline adherence was directly associated with slightly lower functioning at 1 year. However, baseline adherence was associated with better functioning at baseline, and baseline functioning was highly related to better functioning at 1 year. A possible explanation of this counterintuitive finding is that patients with lower adherence at baseline had a greater capacity for improvement during the course of the study. Thus patients with lower adherence at baseline showed greater improvement during the course of the study. In understanding the predictors and also the effects of good adherence, other factors, such as the specific anti-psychotic medication and dose regimen may also be relevant. Treatment dose has been associated to differences in adherence rates [26] and also proper antipsychotic dosing may be necessary for the maximization of treatment outcomes [27].

Several previous studies have also analysed the complex relationship between these variables and their relationship with other clinical factors. Insight has been associated with cognitive impairments in schizophrenia such as social cognition and may also be related to depression [28]. Patients with more metacognitive abilities may also have a better insight, which seems to be independent of neurocognitive deficits [29].

The implications of these findings for patient care are that interventions to enhance medication adherence are particularly appropriate in patients with low illness insight. Specifically, newly developed treatments that have been proven efficacious in improving insight in patients with schizophrenia may lead to better outcomes. For example, the findings that insight may be related to metacognition lead to the development of metacognitive intervention of Lysaker and colleagues [30]. In addition, Van Donkersgoed and colleagues have developed the MERIT (Metacognitive Reflection and Insight Therapy) [31]. Given the association between insight, the therapeutic alliance and medication adherence, specific approaches are needed to improve the therapeutic alliance with the treating team in patients with low insight, as this alliance impacts directly on adherence. Brent has developed a mentalization-based treatment to improve therapeutic relationship [32]. It has also been suggested that interventions to enhance adherence in schizophrenia may be more effective if they focus on treatment-related attitudes, particularly the patient’s perceived necessity for antipsychotic treatment, by exploring and addressing concerns and the patient’s distrust of pharmacotherapy in a more personalized way [14].

### Limitations

A number of limitations should be taken into account when considering the findings of this analysis. Firstly the data were drawn from an observational study. Secondly, assessment of the factors was performed by the same evaluator, which may have increased the inter-relationship of the variables. Thirdly the current analysis included both patients with schizophrenia and those with bipolar disorder. However, the results are similar when conducting separate analyses for patients with schizophrenia and bipolar disorder. Fourth, we have not included medication in the analyses because all patients were taking two different formulations of the same agent and the main report from the original study found no major differences in outcomes between the two formulations [17]. Fifth, we have only analyzed some of the factors that can be related to patient outcomes (for example, clinical subtypes or family and social environment were not analyzed). Sixth, the MARS is a self-reported scale and thus subject to bias. Finally, this was a post-hoc analysis with only one evaluation at follow-up; more data points during the course of the disorder may be necessary to fully understand the relationship between these variables.

## Conclusions

The present study found that insight, therapeutic alliance and adherence are closely related and that all of these factors impact on clinical and functional status in patients with schizophrenia or bipolar disorder. These results highlight the relevance of interventions designed to improve insight and medication adherence. Improvement in insight is likely to lead to increased adherence and improvements in the therapeutic alliance.
